# Deletion of NFKB1 enhances canonical NF-κB signaling and increases macrophage and myofibroblast content during tendon healing

**DOI:** 10.1038/s41598-019-47461-5

**Published:** 2019-07-29

**Authors:** Katherine T. Best, Fredella K. Lee, Emma Knapp, Hani A. Awad, Alayna E. Loiselle

**Affiliations:** 1 Center for Musculoskeletal Research, University of Rochester Medical Center, Rochester, NY 14642 United States of America; 2 Department of Biomedical Engineering, University of Rochester, Rochester, New York United States of America

**Keywords:** Cell biology, Molecular biology

## Abstract

Flexor tendon injuries heal with excessive scar tissue that limits range of motion and increases incidence of re-rupture. The molecular mechanisms that govern tendon healing are not well defined. Both the canonical nuclear factor kappa B (NF-κB) and mitogen activated protein kinase (MAPK) pathways have been implicated in tendon healing. The gene *NFKB1* (proteins p105/p50) is involved in both NF-κB and MAPK signaling cascades. In the present study, we tested the hypothesis that global *NFKB1* deletion would increase activation of both NF-κB and MAPK through loss of signaling repressors, resulting in increased matrix deposition and altered biomechanical properties. As hypothesized, *NFKB1* deletion increased activation of both NF-κB and MAPK signaling. While gliding function was not affected, *NFKB1* deletion resulted in tendons that were significantly stiffer and trending towards increased strength by four weeks post-repair. *NFKB1* deletion resulted in increased collagen deposition, increase macrophage recruitment, and increased presence of myofibroblasts. Furthermore, *NFKB1* deletion increased expression of matrix-related genes (*Col1a1*, *Col3a1*), macrophage-associated genes (*Adgre1*, *Ccl2*), myofibroblast markers (*Acta2*), and general inflammation (*Tnf*). Taken together, these data suggest that increased activation of NF-κB and MAPK via *NFKB1* deletion enhance macrophage and myofibroblast content at the repair, driving increased collagen deposition and biomechanical properties.

## Introduction

Tendons are a dense connective tissue primarily composed of type I collagen. The collagen fibrils are organized into a hierarchical structure that confers strength and enables the tendon to transmit forces from muscle to bone. Following an acute tendon injury, large quantities of disorganized type III collagen-rich scar tissue are transiently deposited at the injury site. The healing process proceeds through inflammation, extracellular matrix deposition, and incomplete remodeling of scar tissue, leading to long-term deficits in tendon function and strength. It is postulated that the initial inflammatory stage of tendon healing drives scar tissue deposition. For example, fetal tendons heal in a regenerative manner following injury and are characterized by minimal inflammation^1,2^. In contrast adult tendons heal via deposition of scar tissue and experience a robust inflammatory phase characterized by an influx of inflammatory cells^3^. However, there is insufficient understanding of the pro-inflammatory signaling pathways that are activated following acute tendon injury, including how these pathways may modulate scar formation during healing.

Canonical nuclear factor kappa B (NF-κB) signaling is a pro-inflammatory signaling pathway that regulates hundreds of different genes including pro-inflammatory cytokines, chemokines, adhesion molecules, and enzymes through the heterodimer p65-p50^4^. Canonical NF-κB signaling has been implicated in both chronic and acute tendon injuries^5,6^. Mitogen-activated protein kinase (MAPK) has also been implicated in scar-mediated tendon healing. MAPK signaling is a varied, multi-tiered signaling cascade with multiple effectors and downstream gene targets^7,8^. The extracellular signal-regulated protein kinases ERK1/2 is a well-characterized MAPK pathway that drives cell proliferation, differentiation, and expression of cell cycle proteins^9^. It has previously been shown that inhibition of ERK2 decreased tendon scar tissue formation following an acute flexor tendon injury and repair^10^.

The *NFKB1* gene encodes the protein p105, which can undergo proteasomal processing to p50^11^. The protein p50 most commonly binds to p65 and together constitute the classic canonical NF-κB heterodimer that drives expression of pro-inflammatory genes. Both p65 and p50 have DNA binding domains; however, only p65 has a transactivation domain, preventing p50 from initiating gene expression. Additionally, it has been shown that p50 can bind to other p50 proteins to form p50 homodimers. The p50 homodimer can bind to DNA, but is unable to initiate gene transcription, therefore acting as a transcriptional repressor. Protein p105 binds to the kinase TPL2, preventing downstream phosphorylation of ERK1/2. Thus, deletion of the *NFKB1* gene results in a complete absence of repressive p50 homodimers, and an uninhibited TPL2 kinase, likely stimulating increased activation of both canonical NF-κB signaling and ERK1/2 signaling.

In the present study, we characterized tendon healing in *NFKB1* wildtype (*NFKB1*^WT^), heterozygote (*NFKB1*^Het^), and knockout (*NFKB1*^KO^) mice, and tested the hypothesis that global *NFKB1*^KO^ activates canonical NF-κB and ERK1/2 signaling pathways, resulting in increased scar tissue deposition. We assessed the differences in canonical NF-κB and ERK1/2 signaling activation between genotypes and analyzed tendon gliding function and biomechanics in both uninjured and repaired flexor tendons. In addition, evaluation of repaired tendons identified changes in the cellular environment, matrix deposition, and downstream gene targets of NF-κB and MAPK signaling, which may drive the mechanical phenotypes that were observed during healing. These data provide evidence that increased activation of canonical NF-κB and ERK1/2 signaling, likely resulting from loss of p50 repressors and freeing of the TPL2 kinase, synergistically result in increased extracellular matrix deposition at the injury site, potentially driven by an increased presence of macrophages and myofibroblasts.

## Results

### *NFKB1*^KO^ does not alter baseline tendon gliding function or mechanical properties

**Figure 1:**
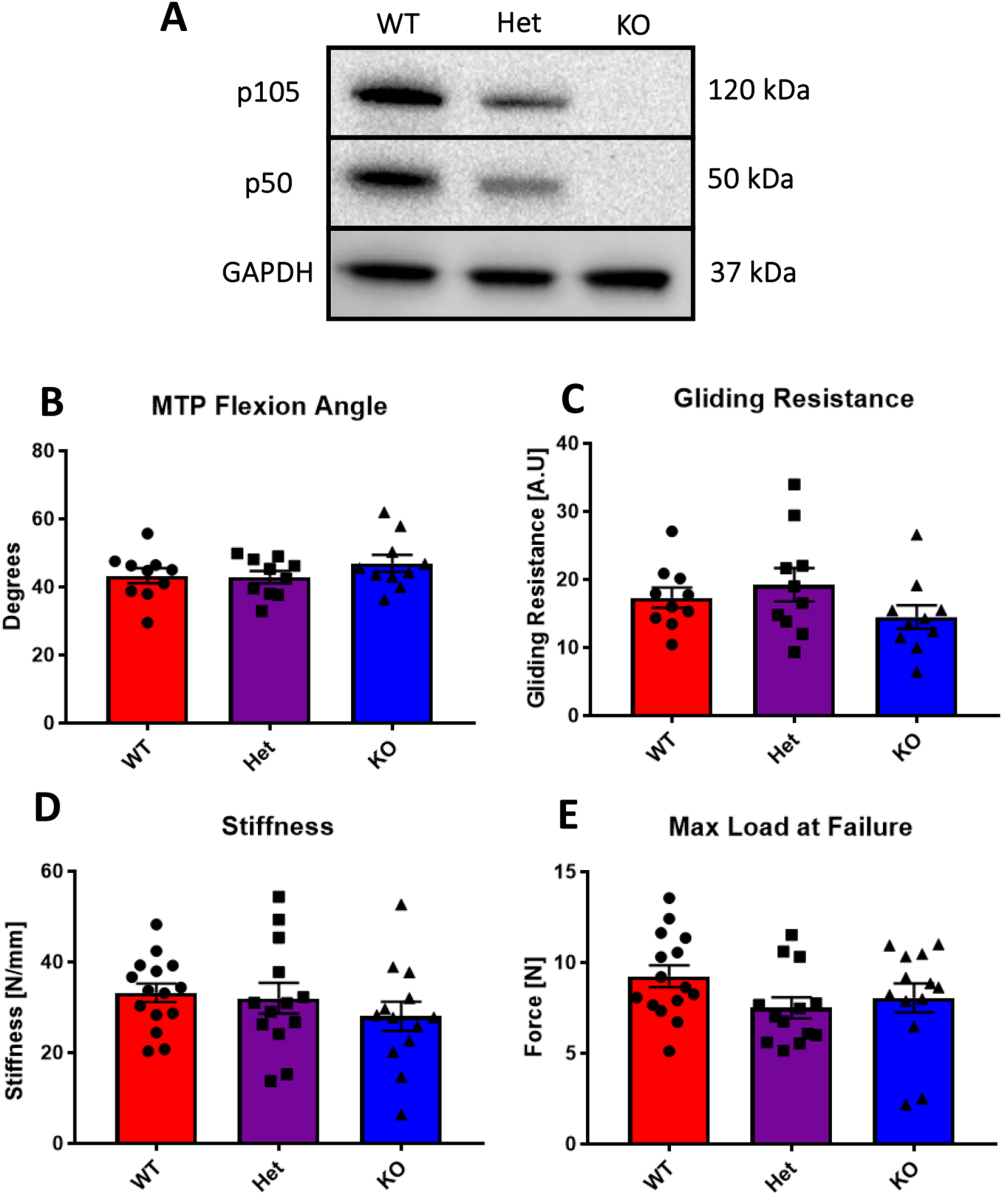
Deletion of *NFKB1* does not affect uninjured tendon gliding function or biomechanical properties. Western blotting for *NFKB1* proteins p105/p50 demonstrates partial and complete depletion in *NFKB1*^Het^ and *NFKB1*^KO^ uninjured tendon, respectively, compared to wildtypes littermates (**A**). Full length blots are presented in Supplementary Fig. 2. Measurement of metatarsophalangeal (MTP) joint flexion angle (**B**), gliding resistance (**C**), stiffness (**D**), and maximum load at failure (**E**) of uninjured wildtype, *NFKB1*^Het^, and *NFKB1*^KO^ tendons showed no significant differences.

Expression of p50 and p105 were substantially reduced in *NFKB1*^Het^ mice, relative to *NFKB1*^WT^, while complete deletion of p50 and p105 were observed in *NFKB1*^KO^ mice via western blot (Fig. 1A). To ensure that *NFKB1*^KO^ did not impair baseline tendon function and mechanical properties, uninjured tendons were assessed. No changes in MTP flexion angle (Fig. 1B), gliding resistance (Fig. 1C), stiffness (Fig. 1D), or maximum load at failure (Fig. 1E) were detected between genotypes.

### *NFKB1* knockout animals exhibit increased activation of canonical NF-κB and MAP kinase signaling during tendon healing compared to WT

**Figure 2:**
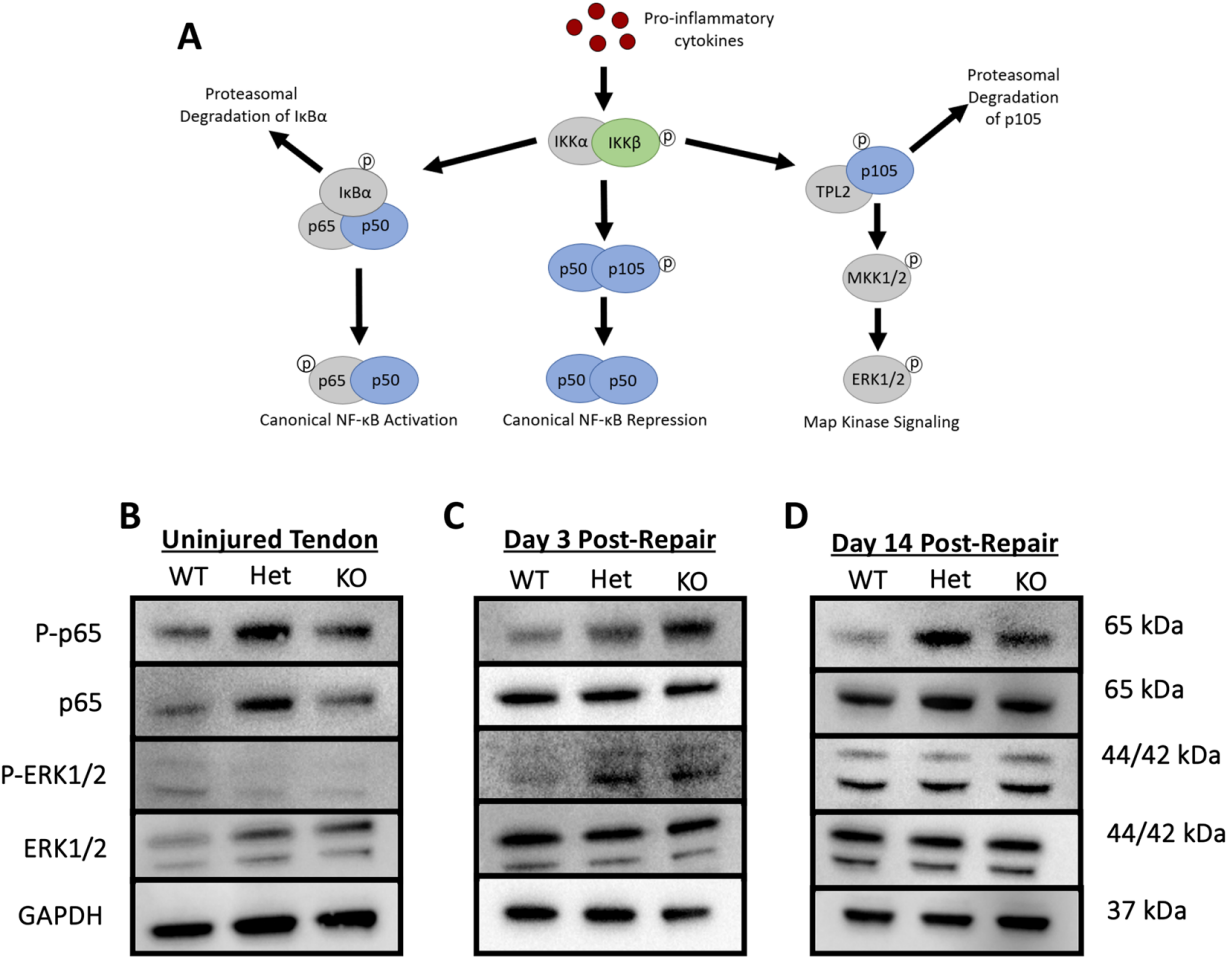
Deletion of *NFKB1* increases activation of canonical NF-κB signaling post-repair. *NFKB1* proteins p105 and p50 are involved in both canonical NF-κB and MAPK signaling cascades (**A**). Western blots to assess canonical NF-κB (p-p65) and MAPK (p-ERK1/2) activation in uninjured (**B**), 3-(**C**) and 14-days (**D**) post-repair in *NFKB1*^*WT*^, *NFKB1*^Het^, and *NFKB1*^KO^. Blots were then stripped and re-probed for total p65, total ERK1/2, and GAPDH (**B**). Full length blots are presented in Supplementary Figs 3–5.

The *NFKB1* gene is involved in both canonical NF-κB and MAP Kinase signaling pathways (Fig. 2A). To assess the differences in pathway activation between genotypes in uninjured and at days 3 and 14 post-repair, western blots were performed. Elevated levels of phospho-p65 (p-p65) and total p65 were detected in *NFKB1*^Het^ uninjured tendon relative to *NFKB1*^WT^ and *NFKB1*^KO^ uninjured tendon (Fig. 2B). Additionally, uninjured *NFKB1*^Het^ and *NFKB1*^KO^ tendons exhibited increased levels of total ERK1/2 protein relative to *NFKB1*^WT^ littermates (Fig. 2B). Increased presence of p-p65 and p-ERK1/2 protein was detected in *NFKB1*^KO^ animals compared to *NFKB1*^WT^ littermates at 3 days post-repair, indicative of increased NF-κB and MAP Kinase activation, respectively (Fig. 2C). *NFKB1*^Het^ animals also exhibited increased p-ERK1/2 and a modest increase in p-p65 (Fig. 2C), suggesting increased NF-κB and ERK signaling in *NFKB1*^Het^ relative to *NFKB1*^WT^. By 14 days post-repair, both *NFKB1*^Het^ and *NFKB1*^KO^ repairs continue to exhibit increased levels of p-p65 relative to *NFKB1*^WT^. However, *NFKB1*^Het^ repairs exhibit more p-p65 than *NFKB1*^KO^ animals, suggesting that *NFKB1*^Het^ had the highest activation of NF-κB signaling by this time-point (Fig. 2D). At day 14 post-repair, there were no apparent differences in p-ERK1/2 between genotypes, which suggests *NFKB1*-independent activation of ERK1/2 signaling at this time point (Fig. 2D). Thus, higher activation of canonical NF-κB signaling was seen in both *NFKB1*^Het^ and *NFKB1*^KO^ tendons relative to *NFKB1*^WT^ at both days 3 and 14, while ERK1/2 signaling was more highly activated in these groups at day 3 alone.

### *NFKB1*^KO^ does not impair gliding function but delays restoration of mechanical properties of healing tendons

**Figure 3:**
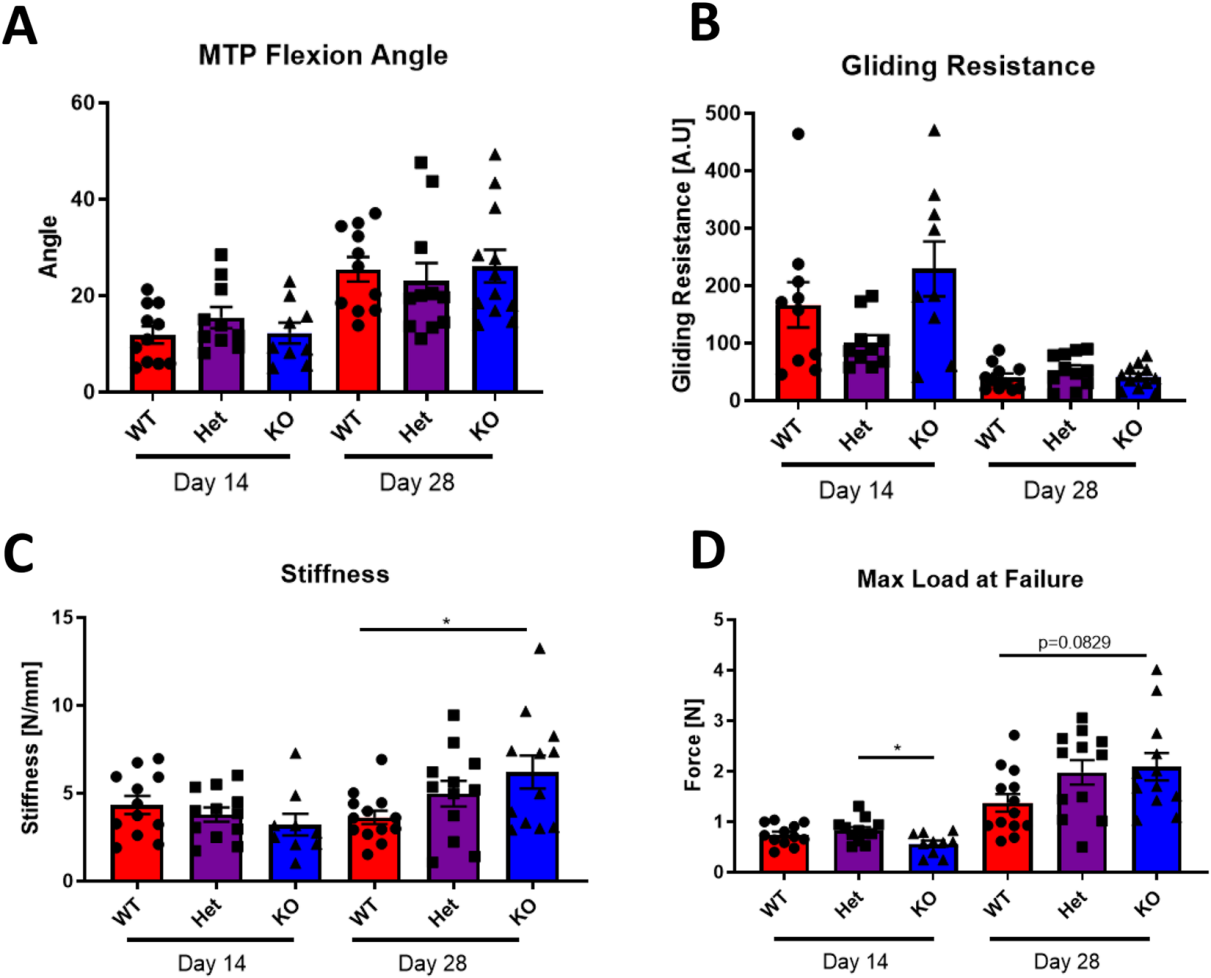
*NFKB1* deletion accelerates late-stage tendon healing. Measurement of metatarsophalangeal (MTP) joint flexion angle (**A**), gliding resistance (**B**), stiffness (**C**), and maximum load at failure (**D**) of 14- and 28-day post-repair wildtype, *NFKB1*^Het^, and *NFKB1*^KO^ tendons. *indicates p < 0.05.

*NFKB1*^KO^ healing tendons did not differ significantly in MTP flexion angle (Fig. 3A) or gliding resistance (Fig. 3B) compared to *NFKB1*^Het^ and *NFKB1*^WT^ littermates at either 14- or 28-days post-repair (Normalized to uninjured data in figure 1– MTP flexion angle: WT^D14^: 27.3%, WT^D28^: 58.9%, Het^D14^: 35.9%, Het^D28^: 53.6%, KO^D14^: 26.0%, KO^D28^: 55.5%) (Normalized to uninjured data in figure 1– Gliding Resistance: WT^D14^: 1225.4%, WT^D28^: 235.3%, Het^D14^: 521.4%, Het^D28^: 277.6%, KO^D14^: 1580.6%, KO^D28^: 284.8%). While the stiffness did not significantly differ between genotypes at 14 days post-repair, *NFKB1*^KO^ tendons were significantly stiffer than *NFKB1*^WT^ tendons (WT: 3.62N/mm ± 0.39, KO: 6.20N/mm ± 0.94, p = 0.0359) at 28 days post-repair, with no significant difference compared to *NFKB1*^Het^ tendon (KO vs Het, p = 0.4572) (Fig. 3C) (Normalized to uninjured data in figure 1– Stiffness: WT^D14^: 13.0%, WT^D28^: 10.8%, Het^D14^: 11.9%, Het^D28^: 15.6%, KO^D14^: 11.4%, KO^D28^: 22.1%). *NFKB1*^KO^ tendon maximum load at failure was significantly decreased compared to *NFKB1*^Het^ mice (Het: 0.826N ± 0.06, KO: 0.562N ± 0.06, p = 0.0210), but not *NFKB1*^WT^ mice (WT: 0.744N ± 0.06, p = 0.1392 vs. KO) at day 14 (Fig. 3D). While the differences in maximum load at failure were not statistically significant at day 28, *NFKB1*^KO^ tendons were trending towards improved strength relative to *NFKB1*^WT^ animals (KO vs WT, p = 0.0829) with no differences compared to *NFKB1*^Het^ mice (KO vs Het, p = 0.9387) (Normalized to uninjured data in figure 1– Maximum Load at Failure: WT^D14^: 8.0%, WT^D28^: 15.2%, Het^D14^: 11.1%, Het^D28^: 26.4%, KO^D14^: 7.0%, KO^D28^: 26.1%). Between days 14 and 28, wildtype tendon maximum load at failure improved by 84% and *NFKB1*^KO^ tendons improved by 272%, indicating accelerated healing for the *NFKB1*^KO^ repairs.

### *NFKB1*^KO^ tendons exhibit increased collagen deposition at the repair site

**Figure 4:**
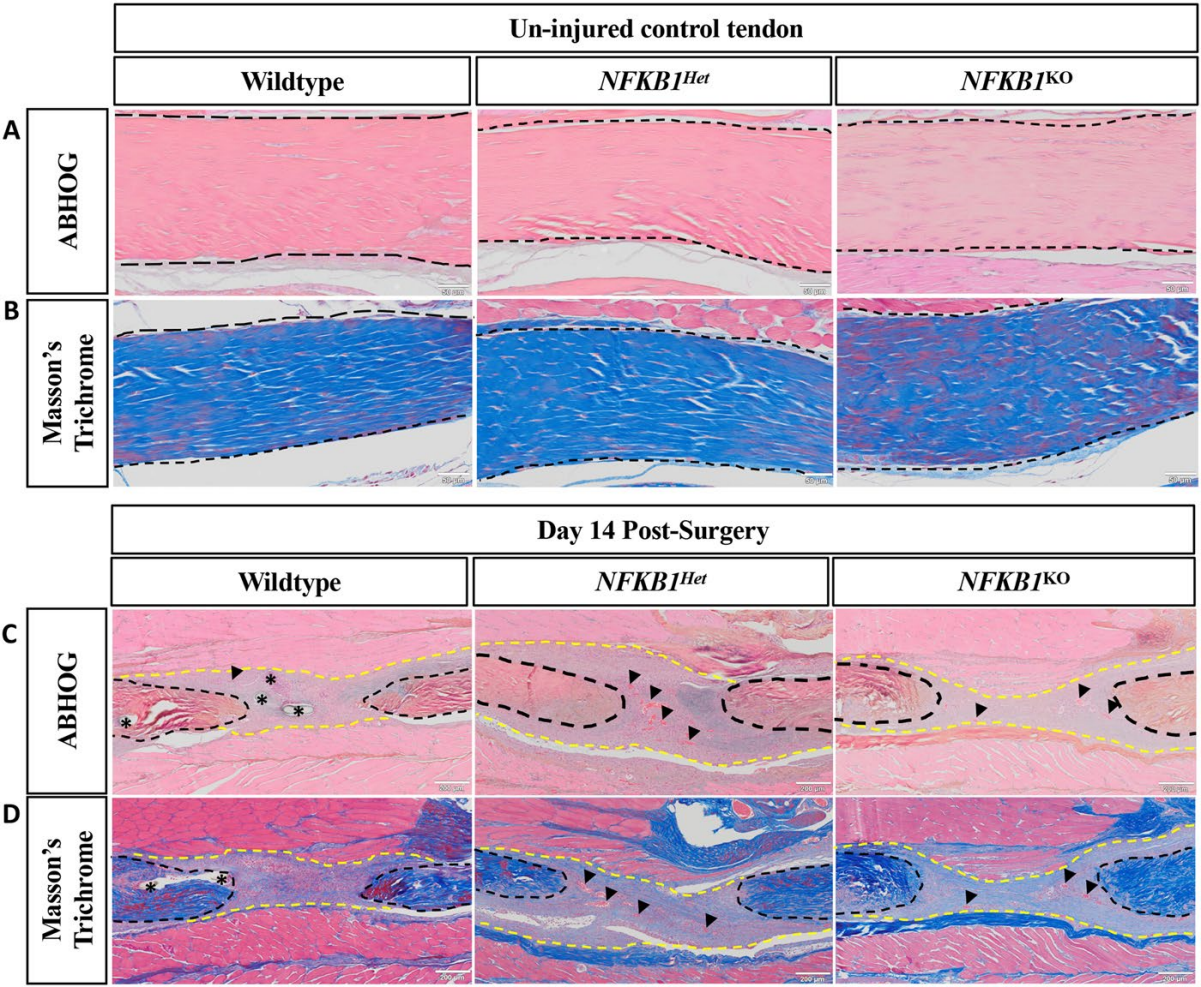
*NFKB1*^Het^ and *NFKB1*^KO^ repairs have increased collagen deposition compared to wildtypes. Histology of *NFKB1*^*WT*^, *NFKB1*^Het^, and *NFKB1*^KO^ tendons uninjured (**A**,**B**) and 14 days post-repair (**C**,**D**). Alcian blue/hematoxylin and Orange G stain demonstrated no obvious differences in scar tissue size or morphology (**C**). Masson’s trichrome stain revealed that *NFKB1*^Het^ and *NFKB1*^KO^ repairs exhibited increased deposition of collagen (**D**, blue stain). Both stains revealed increased presence of blood vessels in *NFKB1*^Het^ and *NFKB1*^KO^ repairs. Tendon is outlined by black dotted lines and scar tissue by yellow dotted lines. Blood vessels are indicated by black arrow heads and sutures by*.

Visualization with ABHOG and Masson’s Trichrome stains exhibited no differences between genotypes in uninjured tendons (Fig. 4A,B). Histologically, there were no apparent differences in scar tissue morphology between genotypes (Fig. 4C). However, *NFKB1*^Het^ and *NFKB1*^KO^ had increased blood vessel presence in the scar tissue compared to *NFKB1*^WT^ mice, possibly indicative of increased angiogenesis. Masson’s trichrome demonstrated that *NFKB1*^KO^ and *NFKB1*^Het^ mice had increased collagen deposition at the repair site relative to *NFKB1*^WT^ littermates (Fig. 4D).

### Increased presence of αSMA+ Myofibroblasts and F4/80+ macrophages at *NFKB1*^Het^ and *NFKB1*^KO^ repair sites

**Figure 5:**
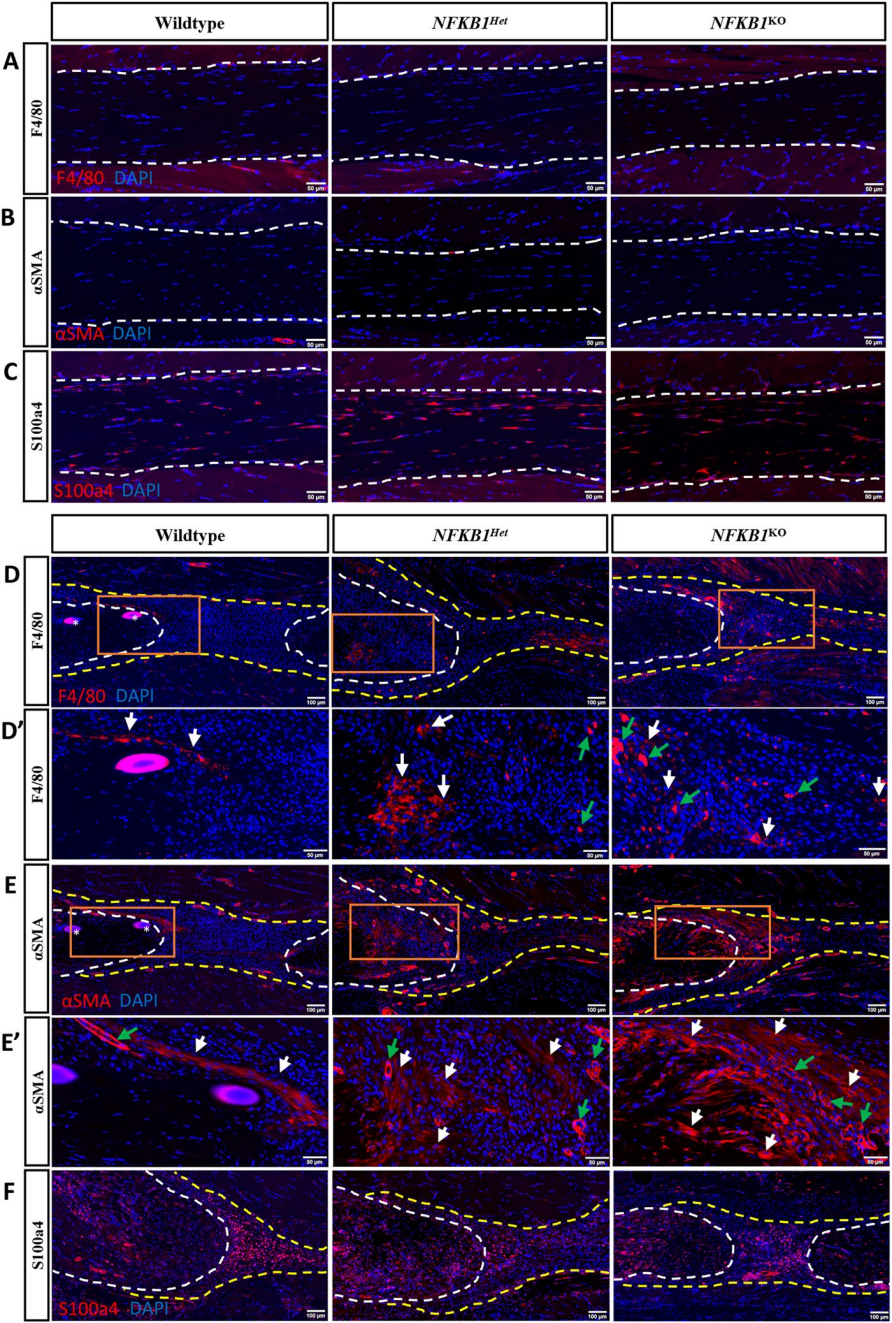
*NFKB1*^Het^ and *NFKB1*^KO^ repairs have increased presence of macrophages and myofibroblasts compared to wildtypes. Immunofluorescence of *NFKB1*^WT^, *NFKB1*^Het^, and *NFKB1*^KO^ uninjured (**A**–**C**) and 14-days post-repair (**D**–**F**). Increased presence of F4/80+ macrophages (**D**,**D’**) and αSMA+ myofibroblasts (**E**,**E’**) present in *NFKB1*^Het^ and *NFKB1*^KO^ repairs relative to wildtypes. No obvious difference in S100a4+ population between genotypes (**F**). Tendon is outlined by white dotted line and scar tissue by yellow dotted line. Orange boxes (**D**,**E**) indicate location of higher magnification images (**D’**,**E**’). Where applicable, examples of positive stain indicated by white arrows, while examples of auto-fluorescent blood cells and α-SMA+ blood vessels indicated by green arrows. Sutures labeled by*.

As it has previously been shown that both macrophages and myofibroblasts contribute to increased matrix deposition and are present during normal flexor tendon healing^12,13^, we used immunohistochemistry to assess their contribution to alterations in biomechanical phenotypes and collagen content seen in *NFKB1*^Het^ and *NFKB1*^KO^ mice. Uninjured tendons contained no F4/80+ or αSMA+ cells, or exhibited changes in S100a4+ cell content, between genotypes (Fig. 5A–C). We observed an increased presence of F4/80+ macrophages and α-SMA+ myofibroblasts in *NFKB1*^Het^ and *NFKB1*^KO^ mice relative to *NFKB1*^WT^ mice at 14 days post-repair (Fig. 5D,E). We have previously shown that S100a4+ cells are present within the tendon and throughout the scar tissue during healing, contributing to the formation of scar^13,14^. There were no observable differences in the S100a4+ population between genotypes 14 days post-repair (Fig. 5F).

### Screening for differential gene expression in *NFKB1*^KO^ animals compared to wildtypes

**Figure 6:**
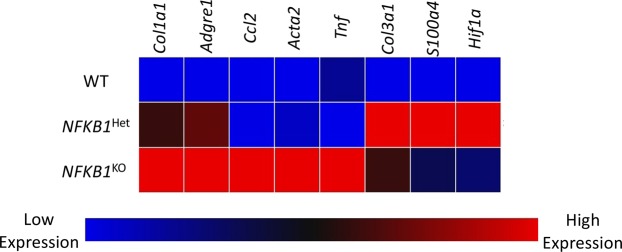
Altered gene expression at 14-days post-repair between wildtype, *NFKB1*^Het^, and *NFKB1*^KO^. mRNA was isolated from wildtype, *NFKB1*^Het^, and *NFKB1*^KO^ repaired tendons 14-days post-repair and gene expression was assessed using a custom PCR array plate (Supplemental Fig. 1). Low gene expression is represented by the blue boxes and high levels of gene expression is represented by the red boxes.

To screen for potential gene targets between genetic groups 14 days post-repair, a custom-designed PCR array plate was utilized (Supplemental Fig. S1). Six genes were found to be upregulated in *NFKB1*^KO^ tendon relative to *NFKB1*^WT^ (*Col1a1*, *Adgre1* (F4/80), *Ccl2*, *Acta2* (α-SMA), *Tnf*, *Col3a1*) (Fig. 6, red color). Five genes were upregulated in *NFKB1*^Het^ relative to *NFKB1*^WT^ (*Col1a1*, *Adgre1*, *Col3a1*, *S100a4*, *Hif1a*) (Fig. 6, red color). Furthermore, five genes were differentially expressed in *NFKB1*^Het^ compared to *NFKB1*^KO^ (*Ccl2*, *Acta2*, *Tnf*, *S100a4*, *Hif1a*) (Fig. 6). Thus, *NFKB1*^Het^ and *NFKB1*^KO^ exhibit divergent gene expression patterns by day 14 post-repair, and both exhibit gene expression patterns different from *NFKB1*^WT^.

## Discussion

In the present study, we assessed the effects of global *NFKB1*^KO^ on flexor tendon healing. We established that loss of *NFKB1* resulted in increased activation of canonical NF-κB and early activation of ERK1/2 signaling following injury, relative to WT littermates. While *NFKB1*^KO^ did not affect baseline tendon gliding function or biomechanical properties, loss of *NFKB1* altered tendon strength during healing. *NFKB1*^KO^ mice exhibited increased collagen deposition, in addition to increased macrophage and myofibroblast presence. Interestingly, while no statistically significant differences in mechanical properties were observed between *NFKB1*^WT^ and NFKB1^KO^ repairs at 14 days post-repair, the maximum load at failure was reduced in *NFKB1*^*KO*^ relative to *NFKB1*^*HET*^ at D14. Consistent with this, complete knockout of p65 in mice contributed to osteoarthritis (OA) development while p65 haploinsufficient animals were protective against OA, suggesting that haploinsufficiency of canonical NF-κB genes can be beneficial in certain pathologies^15^. Thus, loss of one copy of the *NFKB1* gene may be beneficial during early tendon healing, explaining why significant differences were detected between *NFKB1*^KO^ and *NFKB1*^Het^ animals. Furthermore, gene analysis at day 14 post-repair suggested different expression profiles in *NFKB1*^Het^ and *NFKB1*^KO^ animals, which could also explain the differences in day 14 biomechanics at this time point. By 28 days post-repair *NFKB1*^KO^ were no longer significantly different than *NFKB1*^Het^ littermates and were trending towards increased strength relative to *NFKB1*^WT^ littermates. In addition, *NFKB1*^KO^ tendons were significantly stiffer than *NFKB1*^WT^ tendons at 28 days post-repair, suggesting that *NFKB1*^KO^ tendons heal more robustly in later stages of healing compared to *NFKB1*^WT^, perhaps suggesting an accelerated healing process. Previous studies that associate *NFKB1* with improved biomechanical properties are limited; however, downregulation of *NFKB1* gene expression is associated with osteoporotic bone^16,17^, and *NFKB1*^KO^ mice are resistant to bone loss related to mechanical unloading^18^, suggesting that *NFKB1* is necessary for the maintenance of musculoskeletal tissue.

The increase in collagen deposition at the injury site in *NFKB1*^Het^ and *NFKB1*^KO^ repairs relative to *NFKB1*^WT^ at 14 days post-repair is consistent with the increased gene expression of collagens type I and type III, which were also observed in these genotypes. This increase in collagen deposition may in turn contribute to the robust increase in maximum load from 14 to 28 days post-repair in *NFKB1*^*KO*^ repairs. These observations are consistent with increased matrix deposition during skin inflammation and chronic liver injury in *NFKB1*^*KO*^ mice^19,20^. Moreover, several of the cell types present during tendon healing can contribute to collagen-rich scar tissue formation, including fibroblasts, myofibroblasts, and macrophages. Thus, it is possible that *NFKB1*^KO^ may drive excessive scar tissue deposition via changes in recruitment, proliferation, or activation of one or more of these cell types.

Phosphorylation, and therefore activation, of p65 is an accepted marker of canonical NF-κB signaling. NF-κB can drive expression of hundreds of genes, including genes that can continue to re-activate canonical NF-κB signaling (IL-1β and TNF-α) as well as genes that aid in shutting down the pathway (IκBα and p50/p105). Deletion of p50/p105 results in loss of one of the ways canonical NF-κB mediates its own shut-down, resulting in continued, and likely elevated, activation of canonical NF-κB signaling. Thus, deletion of p50/p105 can result in increased canonical NF-κB, and thus increased presence of p-p65. The effects of *NFKB1*^KO^ on both canonical NF-κB signaling and ERK1/2 signaling are cell- and tissue-type dependent. For example, Han *et al*., demonstrate increased NF-κB activation in non-hematopoietic cells and bone marrow derived macrophages of *NFKB1*^*KO*^ mice^21^, while Frantz *et al*. identified decreased NF-κB activation in NFKB1^KO^ spleen lymphocytes^22^. Here we show that *NFKB1*^KO^ increased phosphorylation of p65 during healing, relative to *NFKB1*^WT^, indicating that loss of *NFKB1* results in enhanced activation of canonical NF-κB signaling during tendon healing. Moreover, it is well established that p105 inhibits TPL2 kinase activity, reducing phosphorylation of ERK1/2^11^, and we demonstrate increased early phosphorylation of ERK1/2 at D3 in *NFKB1*^*KO*^, relative to *NFKB1*^WT^. In contrast, Fearn *et al*., demonstrated that *NFKB1*^KO^ prevented ERK1/2 phosphorylation in bone marrow derived macrophages^23^, further exhibiting the cell type-specific effects of *NFKB1*^KO^. In the present study, we found overall increases in canonical NF-κB and early ERK1/2 activation of cells involved in tendon healing, which we hypothesize to be due to the loss of repressive p50 homodimers and uninhibited TPL2 kinase. However, we did not examine the activation of these pathways in specific cell types present at the injury site. Future studies focused on delineating the cell specific contributions of canonical NF-κB and ERK1/2 signaling activation on tendon healing are needed.

NF-κB mediated expression of macrophage-specific chemokines enhances recruitment of macrophages to the healing tissue^24^. Macrophages play many important and diverse roles during healing^25^, and macrophage depletion during tendon healing decreases scar tissue^26,27^. In our study, *NFKB1*^KO^ and *NFKB1*^Het^ mice exhibit increased F4/80+ macrophages presence at the injury site compared to *NFKB1*^WT^ animals. This is further supported by the increased expression of *Adgre1* (F4/80) and *CCL2* (macrophage chemokine) in *NFKB1*^Het^ and *NFKB1*^KO^ repairs. Macrophages can induce fibroblasts to differentiate into myofibroblasts, a population characterized by extracellular matrix deposition and an ability to contract, which aids in wound closure^28^. We found that αSMA+ myofibroblasts cell number appeared elevated in *NFKB1*^KO^ and *NFKB1*^Het^ mice at the repair sites compared to *NFKB1*^WT^ animals, which corresponds to higher expression levels of *ACTA2* in *NFKB1*^KO^ tendons. This is consistent with previous studies that showed *NFKB1*^KO^ animals exhibited elevated scar tissue deposition accompanied by increased αSMA+ myofibroblasts following chronic liver injury^19,20^. Future studies will be needed to elucidate whether the elevated number of αSMA+ myofibroblasts was due to increased recruitment of circulating fibrocytes, increased proliferation of fibroblasts at the repair site that were later induced to differentiate, or an increase in the rate of myofibroblast differentiation. As there are no changes in S100a4+ cells, which are a predominately fibroblastic cell population, the increase in αSMA+ myofibroblast presence seen in *NFKB1*^KO^ repairs is likely due to increased differentiation rather than an overall increase in fibroblast cell number. *NFKB1*^KO^ may modulate the macrophage-myofibroblast axis during healing, resulting in the elevated presence of these populations that could influence the matrix deposition and therefore biomechanical properties of the healing tendon. However, further studies are necessary to delineate the precise effects of each cell type on one another. Altogether, the elevated scar tissue deposition seen in the *NFKB1*^KO^ mice is associated with the increased presence of F4/80+ macrophages and αSMA+ myofibroblasts.

Both canonical NF-κB and MAPK signaling contribute to angiogenesis. NF-κB signaling can drive expression of vascular endothelial growth factor (*VEGF*), which promotes vessel formation^29^, but NF-κB can also drive expression of vascular endothelial grown inhibitor (*VEGI*), which inhibits angiogenesis^30^. ERK signaling can promote both expression and transcriptional activation of HIF-1α, another factor that drives angiogenesis^31^. *NFKB1*^Het^ and *NFKB1*^KO^ mice exhibited increased vessel formation relative to *NFKB1*^WT^ animals (Figs 4C and 5E). *NFKB1*^Het^ tendons also had increased levels of *HIF1A* relative to *NFKB1*^WT^. Increased vascularity likely improves delivery of circulating cells, including monocytes and fibrocytes, to the healing tendon. Monocytes and fibrocytes can differentiate into macrophages and myofibroblasts, respectively, which could explain the increased presence of these cell types in the *NFKB1*^*Het*^ and *NFKB1*^KO^ mice. Macrophages have been shown to release VEGF to stimulate vessel formation, suggesting that an elevated macrophage presence due to increased NF-κB activation could be driving the development of vessel formation^25^.

One limitation of this study is that *NFKB1*^KO^ is a global knockout of *NFKB1* in all cell types. Therefore, it cannot be conclusively stated which cell types specifically contributed to the altered tendon biomechanics, collagen deposition, cell diversity, and gene expression. Further studies are needed to accurately assess how activated NF-κB and ERK1/2 signaling affected the various cell types present at the healing tendon.

Altogether, these data demonstrate that canonical NF-κB and ERK1/2 signaling cascades contribute to tendon healing. Global activation of these pathways resulted in tendon repairs that heal with improved biomechanics relative to wildtype controls. Analysis of *NFKB1*^KO^ repairs suggests this improvement in biomechanics is associated with increased collagen deposition, possibly due to increased macrophage and myofibroblast content at the repair. This study provides further evidence that canonical NF-κB signaling is involved in tendon healing and provides rationale to interrogate the specific NF-κB dependent roles of various cell types during healing, such as tendon cells, macrophages, and myofibroblasts. Understanding the effects of various signaling cascades on tendon healing will help inform on future therapeutic strategies with the goal of minimizing scar tissue formation while improving tendon mechanical properties. Ultimately, directly targeting NF-κB signaling as a method for improving tendon healing in patients would likely be detrimental as canonical NF-κB signaling can influence expression of hundreds of different genes, many of which are important for homeostasis. However, better understanding the downstream processes mediated by NF-κB signaling could reveal candidate pathways that could be viably targeted by therapeutics.

## Methods

### Animal ethics

This study was carried out in strict accordance with the recommendations in the Guide for the Care and Use of Laboratory Animals of the National Institutes of Health. All animal procedures were approved by the University Committee on Animal Research (UCAR) at the University of Rochester.

### Mice

*NFKB1*^KO^ mice were obtained from Jackson Laboratory (#002849, Bar Harbor, ME). *NFKB1*^KO^ ^32^ mice were generated by disrupting exon 6 of the *NFKB1* gene, resulting in loss of *NFKB1* gene expression and the gene products p105 and p50. Animals were maintained as heterozygotes, resulting in litters consisting of wildtype (*NFKB1*^WT^), heterozygote (*NFKB1*^Het^), and knockout (*NFKB1*^KO^) pups. Animals were group-housed with up to five animals per cage in pathogen-free housing with ad libitum access to food and water. All mouse studies were performed with 10–12 week-old male and female mice.

### Flexor tendon repair

To mimic acute tendon injuries repaired in the clinic, mice underwent complete transection and repair of the flexor digitorum longus (FDL) tendon in the right hindpaw as previously described^33^. Briefly, mice were injected prior to surgery with 15–20 μg of sustained-release buprenorphine. Mice were then anesthetized with Ketamine (60 mg/kg) and Xylazine (4 mg/kg). Following preparation of the surgical site, the FDL was transected at the myotendinous junction in the calf to protect the repair site from rupture due to transmitted force. The skin was closed with a 5–0 suture. A small incision was made to the posterior surface of the hindpaw. The soft tissue was retracted and the FDL isolated using forceps, and the FDL was completely transected using micro-scissors. Following FDL transection, the tendon was repaired using 8-0 sutures and the skin closed with 5-0 sutures. Following repair surgery, the animals were unrestricted in movement, food intake, and water consumption.

### Protein extraction and western blot

Total protein was extracted from *NFKB1* mouse tendons uninjured and at days 3 and 14 post-repair. Samples were collected at the repair site and 1–2 mm of native tendon on either side of the repair, with three tendons pooled per genotype per timepoint. Thus, the protein contribution is reflective of both tendon and the surrounding scar tissue. Tendons were homogenized using 0.5 mm zirconium oxide beads and a Bullet Blender Gold Cell Disrupter (Next Advance Inc., Troy, NY), protein was extracted using radioimmunoprecipitation assay buffer (RIPA) buffer with added protease/phosphatase inhibitors, and 20 µg were loaded into each well of a NuPAGE 4–12% Bis-Tris Gel (Invitrogen, Carlsbad, CA). Following transfer, membranes were probed with antibodies for phospho-p65 (1:1000, Cat#: 3033, Cell Signaling, Danvers, MA), total p65 (1:1000, Cat#: 8242, Cell Signaling, Danvers, MA), phospho-ERK1/2 (1:1000, Cat#: 4377, Cell Signaling, Danvers, MA), total ERK1/2 (1:1000, Cat#: 9102, Cell Signaling, Danvers, MA), p105/p50 (1:1000, Cat#: 13586, Cell Signaling, Danvers, MA), and GAPDH (1:1000, Cat#: 2118, Cell Signaling, Danvers, MA).

### RNA extraction and gene array analysis

Total RNA was extracted from healing tendons at 14 days post-repair. RNA was collected from the repair site and 1–2 mm of native tendon on either side of the repair, with three tendons pooled per genotype. RNA was extracted using Trizol reagent (Life Technologies, Carlsbad, CA). 2000 ng of RNA was used for reverse transcription of cDNA using iScript cDNA synthesis kit (Biorad, Cat#:1708890, Hercules, CA). 10 ng of cDNA along with iTaq Universal SYBR Green Supermix (Biorad, Cat#:1725120, Hercules, CA) were added to each well of a custom PCR array plate (Biorad, Hercules, CA) (Supplemental Fig. 1). Data analysis was performed using Biorad CFX Manager 3.1 software (Biorad, Hercules, CA). Of the three housekeeping genes analyzed, 40S ribosomal protein S18 (RSP18) was used as the internal reference gene as it was the most consistent between genotypes. Data is presented as a Clustergram, where red tiles represent high gene expression and blue tiles represent low gene expression.

### Histology and Immunofluorescence

Hindpaws were harvested uninjured and at day 14 post-repair (n = 4–5 per genotype). Hindpaws were fixed in 10% neutral buffered formalin (NBF) at room temperature for 72 hours, decalcified in 14% EDTA (pH 7.2–7.4) for 2 weeks at room temperature, processed, and embedded in paraffin. Three-micron sagittal sections were cut, de-waxed, and dehydrated for analysis. Sections were stained with Alcian blue/hematoxylin and Orange G (ABHOG) for tissue morphology, and Masson’s Trichrome for collagen content. For immunofluorescence, sections were probed with antibodies for α-SMA-CY3 (1:200, Cat#: C6198, Sigma Life Sciences, St. Louis, MO), F4/80 (1:500, Cat#: sc-26643, Santa Cruz, Dallas, TX), and S100a4 (1:2000, Cat#: ab197896, Abcam, Cambridge, MA) overnight at 4 °C. A Rhodamine Red-X AffiniPure secondary antibody was used with F4/80 and S100a4 (1:200, Cat#: 711–296–152, Jackson ImmunoResearch, West Grove, PA). Sections were then counterstained with the nuclear stain DAPI and imaged using a VS120 Virtual Slide Microscope (Olympus, Waltham, MA).

### Assessment of gliding function and biomechanical properties

Tendon gliding function was assessed as previously described^34^. Briefly, hindlimbs were harvested at the knee-joint and the proximal end of the FDL tendon was detached at the myotendinous junction. The FDL tendon was secured between two pieces of tape with cyanoacrylate and the hindlimb was held in an alligator clip. The FDL tendon was loaded incrementally with small weights from 0 to 19 g with images captured after each load. Measurement of the flexion angle of the metatarsophalangeal (MTP) joint relative to the unloaded position were made using Image J. Gliding resistance was derived from the changes in MTP flexion angle over the range of applied loads. An increase in Gliding Resistance and reduction in MTP Flexion Angle is associated restricted range of motion and increased scar tissue. Following gliding testing, the FDL tendon was released from the tarsal tunnel. The proximal end of the tendon and the toes of the hindpaw were help in place by opposing clamps on an Instron 8841 uniaxial testing system (Instron Corporation, Norwood, MA). The tendon was loaded until failure at a rate of 30 mm/minute^34,35^. Nine-15 samples per genotype per time point were analyzed.

### Statistical analysis

Quantitative data was analyzed via GraphPad Prism and is presented as mean ± standard error of the mean (SEM). Normality was assessed using the D’Agostino & Pearson test. For normal datasets, a one-way analysis of variance (ANOVA) with Tukey’s multiple comparisons test was used to analyze gliding and biomechanical data between genotypes at a given time point. For non-normal data (D14 gliding resistance and D14 stiffness), a Kruskal-Wallis test with Dunn’s multiple comparisons test was utilized.

## Supplementary information

Supplementary Info

## Data Availability

All data generated or analyzed during this study are included in this article.
