# Supplementary material for: Deletion of NFKB1 enhances canonical NF-κB signaling and increases macrophage and myofibroblast content during tendon healing
Source: Sci Rep. 2019 Jul 29;9:10926. doi: 10.1038/s41598-019-47461-5 (PMC6662789; doi:10.1038/s41598-019-47461-5)

Supplementary Information

Deletion of NFKB1 enhances canonical NF-κB signaling and increases macrophage and myofibroblast content during tendon healing

Katherine T. Best, Fredella K. Lee, Emma Knapp, Hani A. Awad, Alayna E. Loiselle

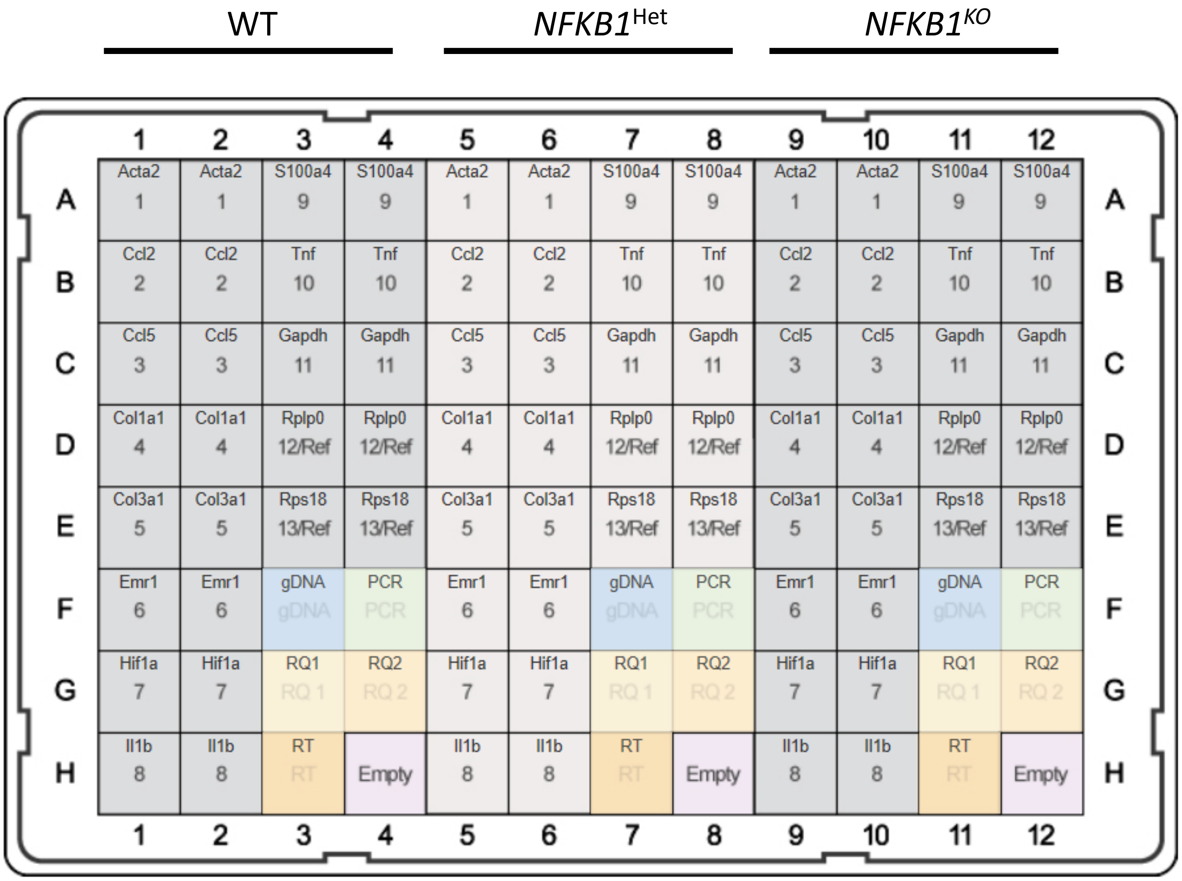

Emr1 is also known as *Adgre1* and is referred to as such in text.

Supplemental Figure 1. Custom PCR Array Plate. Custom Biorad PrimePCR array plate containing genes related to matrix deposition, macrophages, myofibroblasts, and inflammation. Three housekeeping genes (*Gapdh*, *Rplp0*, and *Rps18*) were included.

Full Unedited Gels for Figure 1

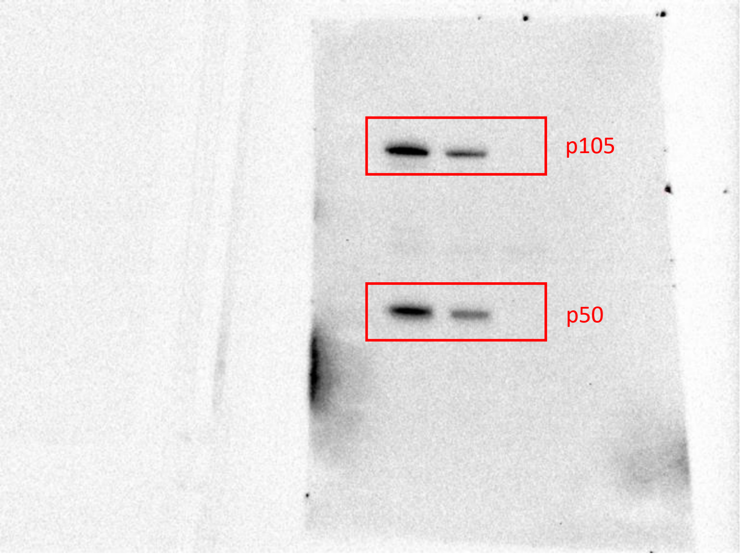

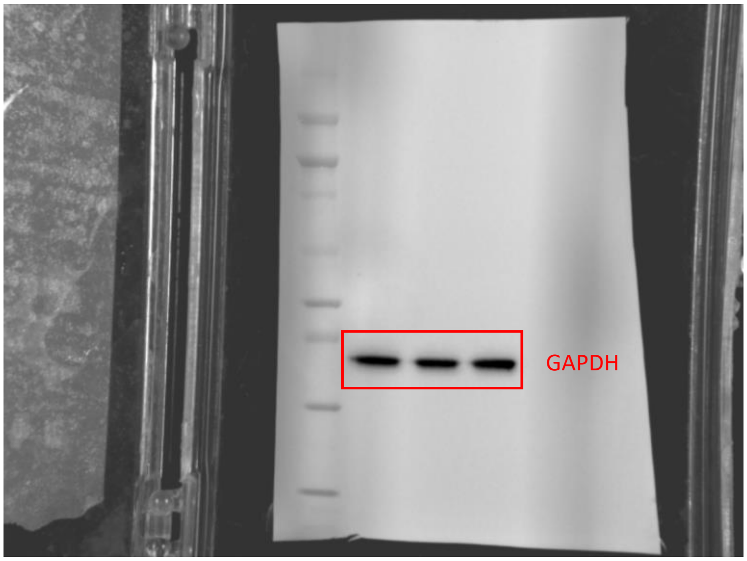

Full Unedited Gels for Figure 2 (Uninjured)

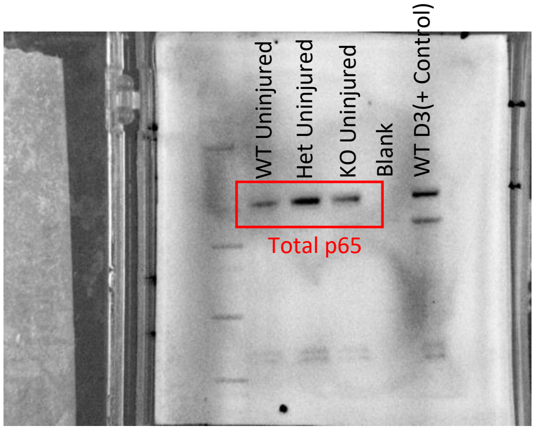

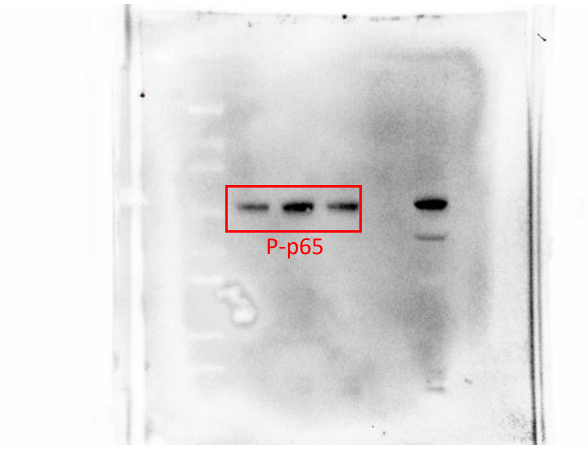

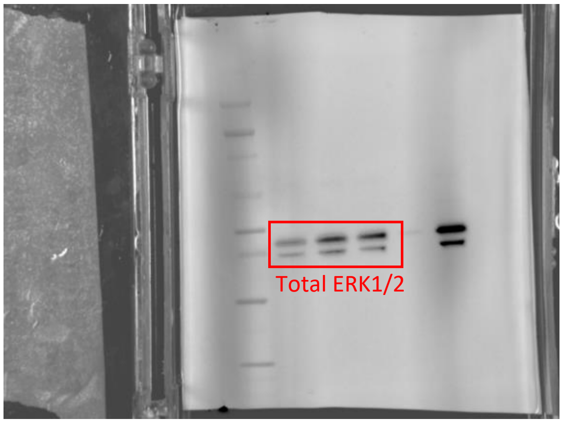

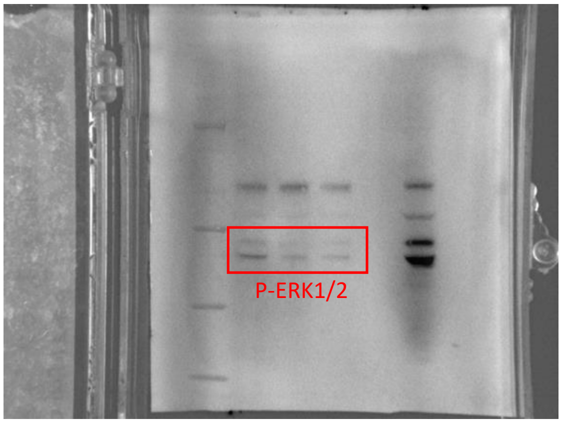

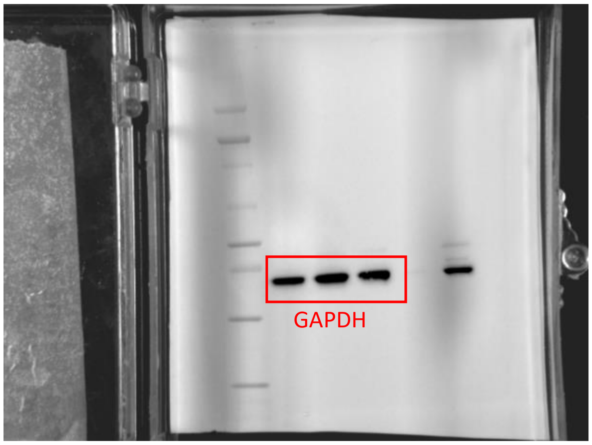

Full Unedited Gels for Figure 2 (Day 3 Repair)

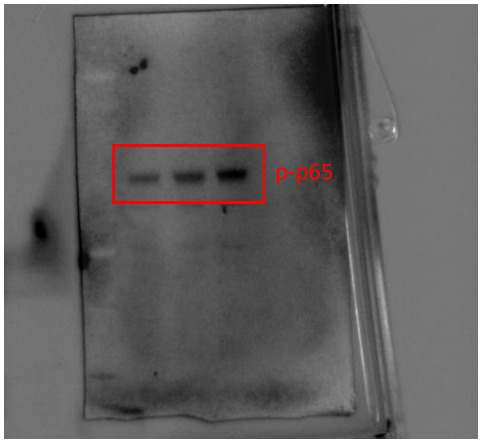

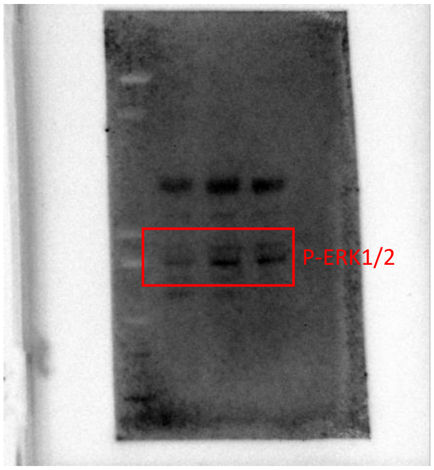

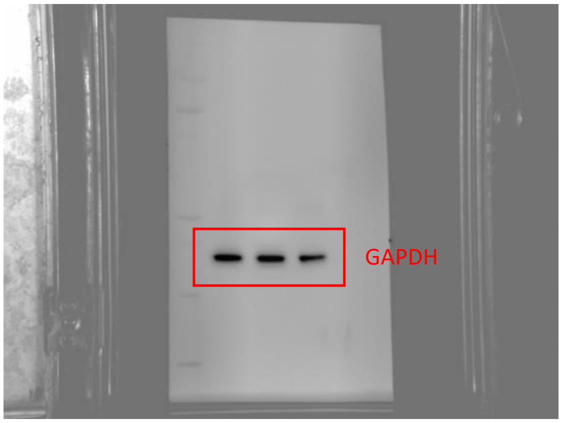

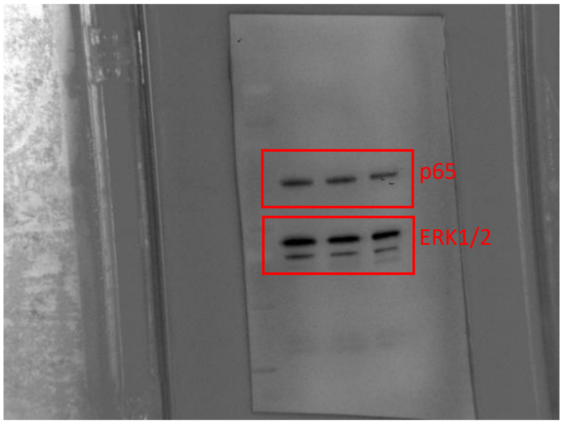

Full Unedited Gels for Figure 2 (Day 14 Repair)

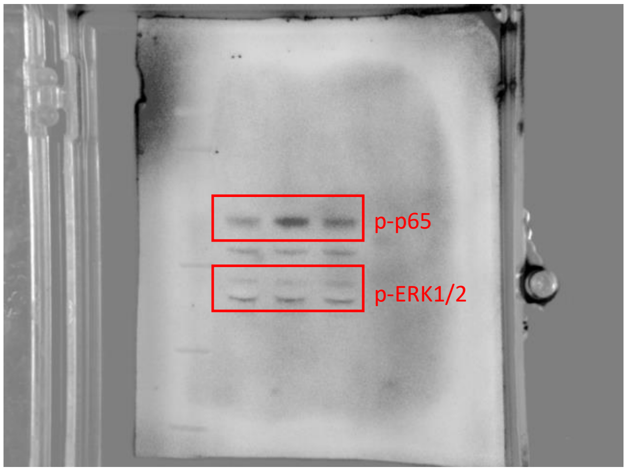

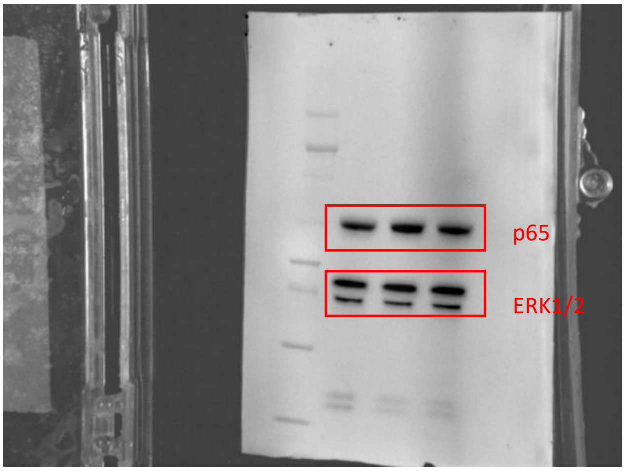

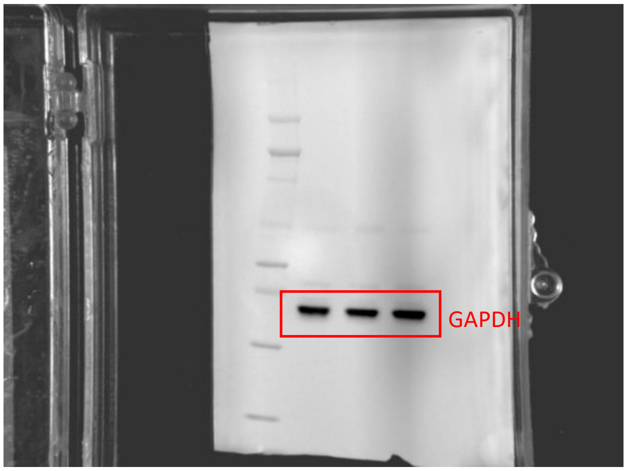

Supplement: Supplementary file 1 — Supplementary Info [file 41598_2019_47461_MOESM1_ESM.pdf]
